# Peer Review of “The Psychological Impact of Hypertension During COVID-19 Restrictions: Retrospective Case-Control Study”

**DOI:** 10.2196/28717

**Published:** 2021-03-30

**Authors:** Dinesh Neupane

**Affiliations:** 1 Johns Hopkins University Baltimore, MD United States

**Keywords:** public health, global health, COVID-19, hypertension, risk, strategy, mental health, behavior, response, anxiety, vaccine, retrospective, perception, prevention, intention

*This is a peer review submitted for the paper “The Psychological Impact of Hypertension During COVID-19 Restrictions: Retrospective Case-Control Study.”*

## Round 1 Review

The paper written by Bonner et al [1], describing the impact of COVID-19 restrictions on people with hypertension, provides important information comparing the current status of risk perceptions, anxiety, and prevention intentions among hypertensive patients compared to healthy controls. The paper is well written, the methods are described well, and the results are presented clearly. I think the manuscript will benefit a lot if the authors consider my comments below.

### Title

I suggest changing the title so that it is clear and informative and reflects the study’s aim and approach. For example: “Risk Perceptions, Anxiety, and Prevention Intentions Among Hypertensive Patient Due to COVID-19 Restrictions in Australia: A Case-Control Study”

### Abstract

Conclusion, second line: Who are vulnerable groups? Have you not reported that there is no difference between cases and controls for the majority of outcome variables? Does this not mean mental health screening should be required for all? I suggest the authors revisit the sentence below in the abstract and conclusion: “…may require targeted psychological screening for vulnerable groups.”

### Methods

1. Is it possible to add a description of the sample size and response rate? This is currently missing in the *Methods* section.

2. Although the reasons for using a linear model, generalized linear model, and ordinal logistic regression are described, it is unclear from the text which test was applied for which estimate. Elaborating on this in the *Methods* section will help readers to understand the methods more appropriately.

### Results

1. Table 1: 42% of participants in the control group indicated that they were taking prescription medicine. What type of medicines were they using? Did you not include healthy controls?

2. Risk perception: What statistical test was applied to calculate the MMD coefficient? I hope you have checked the normality assumptions as the data only have a score range of 0-10. I would be cautious to apply linear regression for such types of data.

3. I suggest adding a table (similar to Table 2) for the follow-up results.

### Discussion

I think the *Discussion* section can be expanded a little bit. The *Results* section has some salient points that warrant discussion. A few suggestions:

- Why is only the willingness to get the influenza vaccine significant? What could be the possible reasons?
- Why is there no statistically significant difference for risk perception? Any literature to support this?
- I think people with hypertension must be more cautious for adopting preventive measures such as social distancing because their mortality and morbidity are often high. However, the results indicate that people with hypertension also have a similar social distancing score. Is this because the score is too high for both groups, or could there be other potential reasons (eg, the same level of access to preventive measures/knowledge, lack of awareness that people with underlying conditions have a high level of mortality, etc)?

### Limitations

Hypertension often presents with other chronic conditions. Including people with other chronic conditions might produce different results since people with both hypertension and other chronic conditions may perceive COVID-19 more severely than people who only have hypertension. The study also has a limitation in terms of residual confounding and long-term impact. I think this has to be reflected in the *Limitations* section.
